# Executive deficits detected in mild Alzheimer's disease using the antisaccade task

**DOI:** 10.1002/brb3.28

**Published:** 2012-01

**Authors:** Liam D Kaufman, Jay Pratt, Brian Levine, Sandra E Black

**Affiliations:** 1 Division of Neurology, Department of Medicine, LC Campbell Cognitive Research Unit, Sunnybrook Research Institute, Sunnybrook Health Sciences Centre, University of TorontoOntario, Canada; 2 Institute of Medical Science, University of TorontoCanada; 3 Department of Psychology, University of TorontoOntario, Canada; 4 Rotman Research Institute, Baycrest University of TorontoToronto, Canada

**Keywords:** Alzheimer's disease, dementia, executive control, saccades

## Abstract

The antisaccade task, a hands- and language-free metric, may provide a functional index of the dorsolateral prefrontal cortex (DLPFC), a region damaged in the later stages of Alzheimer's disease (AD). Our objective was to determine if patients with mild AD made more errors relative to age-matched controls. Thirty patients with mild AD (Mini Mental Status Exam [MMSE] ≥ 17) and 31 age-matched controls completed a laptop version of the prosaccades and antisaccades tasks. Patients with AD made more antisaccade errors, and corrected fewer errors, than age-matched controls. Error rates, corrected or uncorrected, were not correlated with AD MMSE or Dementia Rating Scale scores. Our findings indicate that antisaccade impairments exist in mild AD, suggesting clinically detectable DLPFC pathology may be present earlier than suggested by previous studies.

## Introduction

The antisaccade task has been used increasingly to study Alzheimer's disease (AD) because it provides a parsimonious hands- and language-free measure of dorsolateral prefrontal cortex (DLPFC) function (Kaufman et al. 2010). In the antisaccade task, an eye movement must be directed in the opposite direction from a sudden onset peripheral target (Hallett 1978).

Healthy individuals typically make antisaccade errors (looking toward the target) on 20% of trials, while patients with AD make between 50% and 80% errors (Crawford et al. 2005; Garbutt et al. 2008). Previous studies have included, however, AD patients ranging from mild to severe levels of dementia with mean Mini Mental Status Exam (MMSE) scores between 17 and 21(Currie et al. 1991; Shafiq–Antonacci et al. 2003; Crawford et al. 2005; Garbutt et al. 2008). Reports of a negative correlation between MMSE and antisaccade error rates (low MMSE scores correspond with high error rates) (Currie et al. 1991; Shafiq–Antonacci et al. 2003) suggest that the inclusion of more severely demented patients may have exaggerated the differences in error rates between patients and controls.

Our main objective was to determine whether mild AD patients (MMSE ≥17), make more errors than controls and if so whether error rates correlate with global cognitive measures.

## Methods

### Participants

Sixty-one participants, 30 Patients and 31 community-dwelling age-matched normal volunteers were drawn from the Sunnybrook Dementia Study, a large longitudinal clinical and multimodal imaging study of dementia (Table 1). Patients were diagnosed with probable Alzheimer's Dementia using the NINCDS-ADRDA criteria and the DSM-IV criteria for dementia. Patients with neurological or psychiatric conditions or MMSE scores less than 17 were excluded. A cutoff score of <17 was chosen because 16 represent an inflection point whereby the slope of cognitive decline increases significantly (Feldman et al. 2001). All patients and controls completed an MMSE; additionally the Mattis Dementia Rating Scale (Mattis 1988) was obtained in 19 patients. All participants, or their designated substitute decision maker, provided informed consent for the study, which was approved by the Institutional Research Ethics Board. Although the proportion of female and male participants was unequal between the groups, it is unlikely this affected error rates, as there is no published evidence for sex differences in antisaccade performance (Ettinger et al. 2005).

**Table 1:** Demographics^1^.

	NC	AD	*P*
*N*	31	30	
Sex (female)	18	11	
Age	70.5 (8.2) (50–86)	72.3 (9.7) (51–92)	ns
Years of education	16 (2.6) (11–21)	14.9 (3.3) (10–21)	ns
MMSE	29 (1.1) (26–30)	24.5 (3.2) (17–30)	<0.01
DRS^1^		123.1 (10.3) (102–141)	

1 DRS scores only available on 19 patients in the AD group. Age, Years of Education, MMSE, and DRS values are followed by standard deviation and range.

### Saccade tasks

**Figure 1:**
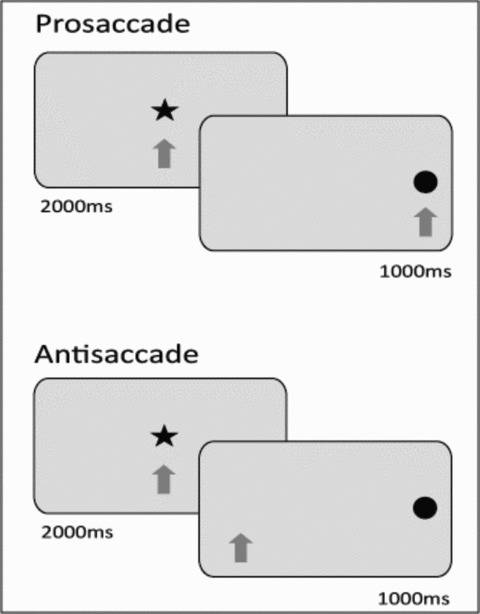
Laptop prosaccade and antisaccade tasks. The fixation star (75 pixels) disappeared simultaneous to the appearance of a peripheral target (75 pixels), 500 pixels left or right of center. Stimulus was presented on a Dell Inspiron 1520 Notebook with a 15.4 screen (1440 by 900 pixels) and participants were recorded with the Notebook's integrated 2.0 M pixel webcam.

The timing and stimulus of both the prosaccade task and the antisaccade task were identical, the tasks only differed in the instructions given to the participant prior to each block. Each participant first completed one block of prosaccades and then two blocks of antisaccades (24 pseudorandom trials per block) (Fig. 1). The step paradigm, in which the central fixation point disappears in synchrony with the peripheral target's appearance, was chosen because (1) it represents a temporal compromise between the gap and overlap paradigms, and (2) because fewer errors are typically made during the step paradigm, relative to the gap paradigm, it was presumed that elderly controls and AD patients would be less frustrated and more compliant. Although the distance between the center and the peripheral target was held constant during each trial, participants were able to move their head freely; thus the visual angle of the offset was not equal for each participant. To demonstrate an understanding of the antisaccade task, prior to the first block, participants first had to successfully point to the location where they were supposed to look for three consecutive trials (Connolly et al. 2000). Instructions were repeated in between blocks. A laptop-integrated web camera recorded the participants'; actions at 30 frames/sec.

### Saccade coding

**Figure 2:**
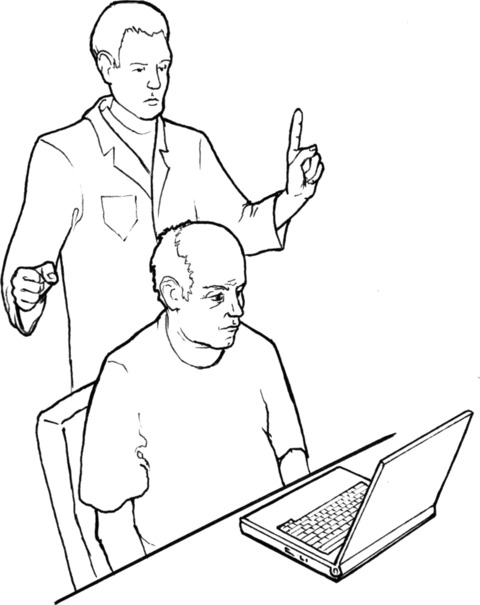
Experimental setup. During the laptop version, the experimenter stood behind the participant and raised a right or left index finger when the participant was suppose to gaze right or left respectively, thus providing a method by which observers of the web camera videos could determine if the participant was looking in the correct direction.

See Figure 2 for experimental setup. If the participant fixated centrally for at least two video frames, then made a saccade in the correct direction two frames, after the experimenter raised a finger and prior to the next trial, the response was coded as correct. It is important to note that the frame rate was variable (20–30 frames per second) for each video and was chosen dynamically by the web camera software; thus, two video frames for one video would be of slightly different temporal length than two frames of another video. Two frames were chosen to (1) ensure that participants followed instructions and returned their gaze to center after each trial and (2) determine that their eyes were not in motion. If they failed to fixate centrally before the next trial, their response was coded as a fixation error. Errors that were corrected before the next trial were coded as corrected errors, while those left uncorrected were coded as uncorrected. Trials in which no action was made were coded as omissions. Fixation and omission errors were excluded from the analysis of antisaccade errors and were analyzed separately. Percentage of errors was defined as: (corrected + uncorrected errors)/(no. of trials) × 100. The correlation between results obtained by the main rater (LDK) and a second rater (CA), who coded videos from 20 participants (10 AD and 10 controls), was 0.88 (*P* < 0.001) indicating a high reliability for coding criteria.

### Statistical analysis

All statistical analysis was completed with SPSS v 16.0 (SPSS, Chicago, IL). Group comparisons were completed on each of the demographic variables and saccade variables using one-way analysis of variance (ANOVA). When the ANOVA assumption of equal variances was not met (e.g., prosaccade errors, antisaccade: error rates, corrections, fixations, and omissions), the Welch's robust tests of equality was utilized (Welch 1947). To assess antisaccade error rates in milder levels of AD, additional analysis was conducted on the subgroup with MMSE scores >22 and another subgroup with MMSE scores >24. The first subgroup, with MMSE scores >22 was selected to provide a direct comparison with the study of Boxer and colleagues (Boxer et al. 2006), while the second group (MMSE >24) was selected because an MMSE score of 24 is usually considered a cutoff point for dementia. Sensitivity, specificity, positive predictive value, and negative predictive value were calculated to assess the diagnostic capacity of antisaccade errors, uncorrected errors, and fixation errors. Sensitivity and specificity calculation required binary classification of performance; therefore, antisaccade performance was categorized as impaired (two standard deviations above the normal controls [NC] mean) or unimpaired (under two standard deviations of the NC mean). The effect size of antisaccade error rates was calculated with Cohen's d, (the mean difference in antisaccade errors between the two groups, divided by the pooled standard deviation) (Cohen 1998). Values derived from the Cohen's d test are categorized into effect sizes that are small (0.2–0.5), medium (0.5–0.8), and large (≥0.8) (Cohen 1992).

## Results

Demographic data for the 61 participants showed no significant baseline differences as summarized in Table 1. Performance metrics are summarized in Table 2. Patients with AD not only made significantly more errors on both the prosaccade (*F*_(1,47.6)_= 4.76, *P* < 0.05) and antisaccade tasks (*F*_(1,47.6)_= 24.72, *P* < 0.001), but also left significantly more antisaccade errors uncorrected (*F*_(1,29.5)_= 22.3, *P* < 0.001). During the antisaccade task, patients made significantly more fixation errors (*F*_(1,31.7)_= 23.6, *P* < 0.01) and omission errors (*F*_(1,31.4)_= 8.1, *P* < 0.01) compared with controls. Both subgroups with MMSE scores >22 (*F*_(1,31.6)_= 18.24, *P* < 0.001) and MMSE scores >24 (*F*_(1,22.3)_= 14.5, *P* < 0.01) made significantly more antisaccade errors than NC.

**Table 2:** Antisaccade performance.

	NC (*n*= 31)	AD (*n*= 30)	AD > 22 (*n*= 22)	AD > 24 (*n*= 17)
MMSE	29 (1.1)	24.5 (3.22)*	26.1 (2.0)	26.8 (1.6)
Errors	22 (18.4)	54.0 (30.3)**	53.5 (30.8)**	53.3 (31.1)*
Uncorrected errors	1.2 (3.4)	32.4 (36.0)**	27.6 (33.4)	25.5 (31.0)
Fixation errors	1.8 (3.8)	17.4 (17.2)*	16.2 (16)	14.7 (14.5)
Omission errors	0.4 (1.1)	3.3 (5.5)*	3.2 (5.6)	4.0 (6.1)

Antisaccade errors are represented in percentages and are followed by their standard deviation. Group statistics were only carried out on values that are followed by asterisks (*P < 0.01; **P < 0.001), and are relative to the control group.

Sensitivity and specificity, and cutoff scores are outlined in Table 3. While all of the metrics provided specificities greater than 0.9, sensitivity was low, with uncorrected errors showing the highest sensitivity (sensitivity = 0.63). Prosaccade errors were not included in sensitivity and specificity as the amount of performance overlap between the two groups was large (Cohen's d = 0.56).

**Table 3:** Diagnostic capacity of antisaccade metrics.

	Cutoff	Number of impaired	Sensitivity	Specificity	PPV	NPV	Cohen's d
Errors	>58.8%	15 AD, 1 NC	0.5	0.97	0.94	0.67	1.28
Uncorrected errors	>7.9%	19 AD, 2 NC	0.63	0.94	0.9	0.73	1.22
Fixation errors	>9.5%	16 AD, 2 NC	0.53	0.94	0.88	0.67	1.25
Omissions	>2.7%	9 AD, 2 NC	0.3	0.94	0.82	0.58	0.73

## Discussion

**Figure 3:**
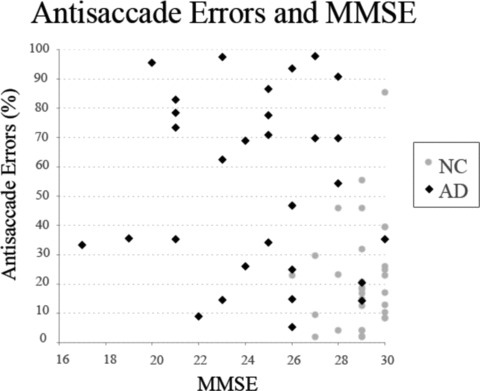
Antisaccade errors and Mini Mental Status Exam (MMSE) scores are plotted on the *x*-axis, while percentage of antisaccade errors are plotted on the *y*-axis. Patients with Alzheimer's disease (AD) and normal controls (NC) are represented by black diamonds and gray circles, respectively.

The current study examined antisaccade performance in patients with mild AD and elderly controls using a novel laptop-based antisaccade task. Patients with AD, even those with MMSE cutoff >24, made significantly more antisaccade errors than controls on both versions of the antisaccade task, and left significantly more errors uncorrected. The effect sizes indicate a large mean magnitude of difference between the two groups, which could be detected in smaller sample sizes. However, despite these large effect sizes in antisaccade performance, sensitivities were low because almost a third of AD patients were unimpaired (Fig. 3). In contrast, antisaccade metrics are highly specific in this study sample, as only two participants in the NC group were impaired. In contrast to other studies (Currie et al. 1991; Shafiq–Antonacci et al. 2003; Boxer et al. 2006), we did not find a correlation between general measures of dementia, such as the MMSE or DRS, and antisaccade error rates.

## Antisaccade Errors Elevated in Mild AD

We hypothesized that previously reported differences in error rates between patients with mild AD and elderly controls were mainly due to the inclusion of more severely demented patients who tend to make 100% errors on the task. To test this hypothesis, we tested AD patients with MMSE scores ≥17 and repeated our analysis on subsets of patients with MMSE scores >22 and greater than 24.

To our knowledge, only the study conducted by Boxer and colleagues (Cohen 1992) has examined antisaccade error rates in mild AD and they did not find a significant difference from elderly controls. They posited that frontal pathology is a late feature in AD and, thus, patients with mild AD would not have “sufficient” pathology to be impaired on the antisaccade task (Boxer et al. 2006). Mild AD is thought to correspond with Braak and Braak's stage 4, a stage in which neurofibrillary changes in the DLPFC are still relatively mild. During Braak and Braak stages 5–6, which are thought to correspond with moderate to severe AD, DLPFC pathology is more evident (Braak and Braak 1991). It would thus be expected that persons with mild AD would have insignificant amounts of DLPFC pathology and would not be impaired on the antisaccade task. However, using a larger sample size, we have shown that about two-thirds of the patients with mild AD do in fact make significantly more errors than controls, implicating sufficient frontal neuropathology to reveal an involuntary control impairment. In fact, there is mounting evidence that executive deficits do occur earlier in disease onset, during a pre-AD stage called mild cognitive impairment and that in vivo amyloid pathology (Pike et al. 2007) and DLPFC structural changes can be detected during MCI and mild AD. For instance using MRI, Mosconi and colleagues (Mosconi et al. 2007) identified a significant degree of DLPFC white matter atrophy in patients with MCI who progressed to AD. Other reports suggest that AD is heterogeneous, with a subset of AD demonstrating pronounced frontal deficits, causing diagnostic confusion with Frontotemporal Degeneration (FTD) (Snowden et al. 2007a), although the self-regulatory disorder is less severe (Snowden et al. 2007b). A large autopsy sample of clinically diagnosed FTD studied by Snowden and colleagues contained only 2% of AD patients with pronounced frontal deficits, but it seems likely that a continuum of DLPFC pathology may exist in AD with some patients having intermediate degrees of frontal dysfunction. As sample sizes become smaller, the probability of capturing the variation in frontal pathology would decrease. Hence, the subset of patients studied by Boxer and colleagues (Boxer et al. 2006) may have been less likely to capture this variation than a study with a larger sample, such as the current study.

## Dementia Severity and Antisaccade Errors

A significant correlation between general measures of dementia, such as the DRS or the MMSE, has been consistently reported, suggesting that error rates, and ultimately DLPFC pathology, might simply be predicted by general levels of dementia. We found that the mean antisaccade error rate of AD patients, 55%, was relatively low compared with previously reported antisaccade error rates of 50–80%. Although this study was not strictly comparable to previous studies, the comparison reveals that the exclusion of more severely demented patients may have resulted in lower mean error rates relative to previous studies, which did include severely demented patients.

We were unable to replicate the previously reported correlations between error rates and MMSE scores within the AD group, likely for several possible reasons. First, the relationship between MMSE and antisaccade error rates in previous studies may have been driven by the more severely demented patients who consistently perform poorly on the antisaccade task, and were excluded for our study. As discussed above, this suggests that antisaccade error rates, and potentially frontal neuropathology, may not reflect overall dementia severity during mild stages of AD. Second, the heterogeneous nature of AD renders the MMSE an unreliable metric for dementia severity. For instance, lower MMSE scores might reflect domain-specific impairments in language or memory, which are heavily weighted in the MMSE, while executive functions remained preserved, or at least are not well captured by the MMSE. The DRS is more weighted for dorsolateral frontal functions but the smaller sample size may have been insufficient to detect correlation. Both possibilities are not mutually exclusive and could contribute to the differences between this study's findings and previous investigations.

We considered the possibility that the group differences were attributable to failure to maintain task instructions over task blocks (Welch 1947). As noted in the methods, subjects were required to point to the correct location where they were supposed to look for three consecutive trials prior to the start of the first block. Instructions were reinforced between blocks. Although loss of task set cannot be ruled out as contributing to our findings, we do not consider this to be explanatory as the patients appeared able to maintain task set for the 72-sec duration of the block, as indicated by their ability to switch instructional set between pro- and antisaccade blocks, even though they were error prone. The low rates of prosaccade errors (3.5% for AD versus 1.9% for NC), although significantly different, also suggests that the AD patients were able to follow the instructions. To conclusively rule out task set maintenance problems, future studies should verify task set instructions before and after each block. Augmenting fixation cues with task set information, further reducing the set maintenance element of the task, could be used as a manipulation check to evaluate set maintenance effects.

## Conclusions

A progressive deficit in episodic memory is the most prominent feature of AD; however, there is an increasing awareness that AD is heterogeneous and even early in the course can be associated with varying degrees of impairment in the visuospatial, executive, and language domains (Buck et al. 1997; Galton et al. 2000; Alladi et al. 2007). Our findings highlight that impairments in an inhibitory control function, manifested by increased antisaccade errors, occur earlier in AD than posited by previous antisaccade studies, and that in mild AD antisaccade errors are not correlated with general measures of dementia such as the MMSE. The findings presented in this study provide further evidence that antisaccade error rates can be easily measured and may potentially provide a clinical method for detecting early frontal dysfunction in AD.
